# Aerobic fitness and body fatness describe minimal variability in the thermoregulatory responses to exercise after accounting for heat production and body size

**DOI:** 10.1186/2046-7648-4-S1-A11

**Published:** 2015-09-14

**Authors:** Matthew Cramer, Ollie Jay

**Affiliations:** 1 School of Human Kinetics, University of Ottawa, Canada; 2 Thermal Ergonomics Laboratory, Faculty of Health Sciences, University of Sydney, Australia

## Introduction

Aerobic fitness (VO_2max_) and body fatness have been regularly suggested as important determinants of core temperature and sweating responses to exercise [3,5], but recent studies suggest that biophysical factors related to heat production (H_prod_), total body mass (TBM), and body surface area (BSA), predominantly influence rectal temperature changes (ΔT_re_) and sweating [1,2,4]. The present study tested the hypotheses that (i) individual variation in ΔT_re_, whole-body sweat loss (WBSL), and steady-state local sweat rate (LSR_ss_) is determined primarily by H_prod _(W.kg^-1 ^TBM), evaporation required for heat balance (E_req_, W), and E_req _(W.m^-2^), respectively, and (ii) factors related to VO_2max _and body fat percentage (BF%) contribute minimally to the residual variance in these responses.

## Methods

Twenty-eight male subjects [TBM: 78.2(11.3) kg, BSA: 1.96(0.15) m^2^, VO_2max_: 3.86(0.68) L.min^-1^)] performed exercise at external workloads corresponding to a wide range of %VO_2max _(32.2-80.0%), H_prod _(5.2-12.1 W.kg^-1 ^TBM), and E_req _(256-672 W) in 24.8(0.7) °C, 33.4(12.2) % RH, and 1.2(0.1) m.s^-1 ^air velocity. T_re _and forearm LSR were measured continuously; WBSL was estimated from changes in body mass. Forward stepwise multiple regression analysis was subsequently performed and partial contributions of each independent variable were determined using standardized regression coefficients.

## Results

H_prod _(W.kg^-1 ^TBM) alone described ~50% of the variance in ΔT_re _(adjusted R^2 ^= 0.496, P < 0.001), while BSA-to-mass ratio and BF% added 4.3% and 2.3%, respectively, to the explained variance. For WBSL, E_req _(W) alone explained ~71% of the variance (adjusted R^2 ^= 0.713, P < 0.001), and the inclusion of BF% explained an additional 2% of the variance in WBSL. Similarly, E_req _(W.m^-2^) correlated significantly with LSR_ss _(adjusted R^2 ^= 0.603, P < 0.001), while %VO_2max _contributed an additional ~4% to the total variance.

## Discussion

Previous findings that identified VO_2max _and body fatness as important modulators of core temperature and sweating may be confounded by collinearity between independent variables, since fitter individuals tend to be lighter and leaner and thus generate more heat (in W.kg^-1 ^TBM) and have a higher E_req _(in W and W.m^-2^) at a fixed %VO_2max_, resulting in expectedly higher T_re _and sweating rates. The relatively minor independent contribution of BF% and %VO_2max _to these responses warrants consideration.

## Conclusion

Biophysical factors related to heat production and body size explained ~54-71% of the total variability in the core temperature and thermoregulatory sweating responses to exercise in a compensable environment, with only a minor contribution (<4%) to the explained variance in ΔT_re _and WBSL by BF%, and LSR_ss _by %VO_2max_.
